# Incidence Trends and Epidemiology of Staphylococcus aureus Bacteremia: A Systematic Review of Population-Based Studies

**DOI:** 10.7759/cureus.25460

**Published:** 2022-05-29

**Authors:** Joya-Rita Hindy, Juan A Quintero-Martinez, Alexander T Lee, Christopher G Scott, Danielle J Gerberi, Maryam Mahmood, Daniel C DeSimone, Larry M Baddour

**Affiliations:** 1 Infectious Diseases, Mayo Clinic, Rochester, USA; 2 Quantitative Health Sciences, Mayo Clinic, Rochester, USA; 3 Library, Mayo Clinic, Rochester, USA

**Keywords:** staphylococcus aureus, bacteremia, trends, population-based study, incidence

## Abstract

Objectives: To determine incidence trends of *Staphylococcus aureus* bacteremia (SAB) from population-based studies from multiple countries.

Methods: A contemporary systematic review was conducted using Ovid Cochrane Central Register of Controlled Trials (1991+), Ovid Embase (1974+), Ovid Medical Literature Analysis and Retrieval System Online (MEDLINE) (1946+ including epub ahead of print, in-process & other non-indexed citations), and Web of Science Core Collection (Science Citation Index Expanded 1975+ and Emerging Sources Citation Index 2015+). Two authors (J.R.H. and J.A.Q.M.) independently reviewed all studies and included those that reported population-based incidence of SAB in patients aged 18 years and older.

Results: Twenty-six studies met inclusion criteria with the highest number (n=6) of studies conducted in Canada. The incidence of SAB ranged from 9.3 to 65 cases/100,000/year. The median age of patients with SAB ranged from 62 to 72 years and SAB cases were more commonly observed in men than in women. The most common infection sources were intravascular catheters and skin and soft tissue infections. SAB incidence trends demonstrated high variability for geographic regions and calendar years. Overall, there was no change in the incidence trend across all studies during the past two decades.

Conclusion: Multiple factors, both pros, and cons are likely responsible for the overall stable SAB incidence in countries included in this systematic review. Some of these factors vary in geographic location and prompt additional investigations from countries not included in the current review so that a more global characterization is defined.

## Introduction and background

*Staphylococcus aureus* is a predominate pathogen in cases of bacteremia in both the community and healthcare settings [1,2], with a mortality rate as high as 30% [3]. The incidence of *S. aureus* bacteremia (SAB) varies depending on risk factors of the studied population and infection control practices in healthcare facilities. Surveillance of SAB trend incidence is crucial to evaluate its impact on public health agencies and to enhance the development of infection prevention and control strategies [4]. Unlike hospital-based studies, population-based studies prevent selection biases and allow standardization of incidence rates for a reference population, making them one of the best tools to assess infectious diseases epidemiology [5]. The most recent systematic review of population-based studies assessing the incidence of bacteremias due to a variety of organisms was conducted in 2013, with incidence rates reported through 2008 [6]. Therefore, we conducted a systematic review of contemporary population-based studies investigating the incidence of SAB from numerous geographic regions during the past two decades as presented herein.

## Review

Methods

Data Sources and Searches

A literature search was done by a medical librarian (D.J.G.) for the concepts of SAB and incidence rates. Search strategies were created using a combination of keywords and standardized index terms. Searches were run on September 2, 2021, in Ovid Cochrane Central Register of Controlled Trials (1991+), Ovid Embase (1974+), Ovid Medical Literature Analysis and Retrieval System Online (MEDLINE) (1946+ including epub ahead of print, in-process, and other non-indexed citations), and Web of Science Core Collection (Science Citation Index Expanded 1975+ and Emerging Sources Citation Index 2015+). After limiting the results based on exclusion criteria (listed below), a total of 26,552 citations were retrieved. Deduplication was performed in Covidence leaving 16,499 citations. Full search strategies are provided in the *appendices*. Two reviewers (J.R.H. and J.A.Q.M.) performed the literature review and any disagreements were solved by a discussion with an additional reviewer (L.M.B.). This review was registered in Open Science Framework (OSF).

Inclusion and Exclusion Criteria

All population-based investigations published in the English language from 2001 through 2020 on SAB incidence trends in the adult population were included in this review. Studies that did not report population-based estimates were not included, such as case reports, single-center, and multi-center studies, clinical trials, conference abstracts, systematic reviews, and studies on animals [7]. Studies conducted that focused on specific sub-groups (e.g., patients with HIV infection or end-stage renal disease [ESRD]) or published in a language other than English were also excluded.

Data Extraction

The following information was extracted from the included studies: first author's last name, year of publication, country of origin (+/- specific region studied), population size, and incidence rate (per 100,000 person-year). Two reviewers (J.R.H. and J.A.Q.M.) extracted data from the studies independently; study investigators were contacted if additional information was needed. The incidence rate of SAB was the primary outcome. Secondary outcomes were age, sex, comorbidities, methicillin resistance of the blood culture isolate, site of onset, and mortality rates.

Statistical Analysis

When available, incidence rate estimates from separate periods within each study were extracted. If estimates from separate periods were not available, overall incidence rate estimates were used. When necessary, these incidence rate estimates were transformed to represent incidence rates per 100,000 population. To visualize the data, incidence rate estimates were plotted by time point and study. For periods where reported incidence rates spanned multiple years, the median of the period was chosen for plotting. To look at trends across the entire period of interest, a simple linear regression model was fit to the individual estimates to help visually interpret the overall trend over time. The SAS, version 9.4 (SAS Institute, Cary, NC, USA) was used for plotting and regression.

Results

After removing all duplicates, a total of 16,499 citations were identified from the search engines (supplemental data) and their abstracts were screened. Thirty-eight of them were chosen for full-text review and 26 studies met inclusion criteria (Table 1).

**Table 1:** Incidence rates of SAB reported in the 26 included population-based studies. SAB: *Staphylococcus aureus* bacteremia *This study included only community-onset SAB

First author, publication year	Country (+/- region)	Years	Population	SAB/10^5^/year
Mejer et al., 2012 [8]	Denmark	1995–2008	5,350,000	22.7
Nielsen et al., 2014 [9]	Denmark, Funen County	2000–2008	390,000	27.3
Thorlacius-Ussing et al., 2019 [10]	Denmark	2008–2015	5,475,791	24.9
Wilson et al., 2011 [11]	England	2004–2008	61,500,000	21.8
Lyytikäinen et al., 2005 [12]	Finland	1995–2001	5,000,000	14
Skogberg et al., 2012 [13]	Finland	2004–2007	5,300,000	20
Jokinen et al., 2018 [14]	Finland, Pirkanmaa County	2005–2015	543,700	31.5
Asgeirsson et al., 2011 [15]	Iceland	1995–2008	300,000	24.5
Asgeirsson et al., 2011 [16]	Iceland	2003–2008	300,000	29
Mehl et al., 2017 [17]	Norway, Nord-Trøndelag County	2002–2013	72,000	25
Blomfeldt et al., 2016 [18]	Norway, Oslo	2011–2014	500,000	27.6
Ruiz-Azcona et al., 2020 [19]	Spain, Valencia	2013–2017	5,000,000	9.3
Allard et al., 2008 [20]	Canada, Quebec	1991–2008	445,000	27.6
Laupland et al., 2013 [21]	Canada, Victoria	1998–2005	750,000	15.5
^*^Laupland et al., 2007 [22]	Canada, Calgary	2000–2004	1,000,000	13.5
Laupland et al., 2008 [23]	Canada, Calgary	2000–2006	1,000,000	19.7
Lam et al., 2019 [24]	Canada, Calgary	2012–2014	1,288,000	27.8
Laupland et al., 2021 [25]	Canada, British Columbia	2010–2020	191,000	31.9
Morin et al., 2001 [26]	USA, Connecticut	1998	1,124,337	20.9
El Atrouni et al., 2009 [27]	USA, Minnesota, Olmsted County	1998–2005	124,277	38.2
Hindy et al., 2022 [28]	USA, Minnesota, Olmsted County	2006–2020	164,365	31.1
Tong et al., 2009 [29]	Australia, Northern territory	2006–2007	176,000	65
Tong et al., 2012 [30]	Australia	2007–2010	21,750,000	11.2
Mitchell et al., 2012 [31]	Australia, Tasmania	2009–2010	503,292	21.3
Huggan et al., 2010 [32]	New Zealand, Canterbury	1998–2006	478,000	21.5
Laupland et al., 2013 [33]	Australia, Canberra, Queanbeyan, and New South Wales; Canada, Sherbrooke (Quebec), Victoria (British Columbia), and Calgary (Alberta); Denmark, North Denmark; Finland; Sweden, Skaraborg County	2000–2008	83,000,000	26.1

**Figure 1:**
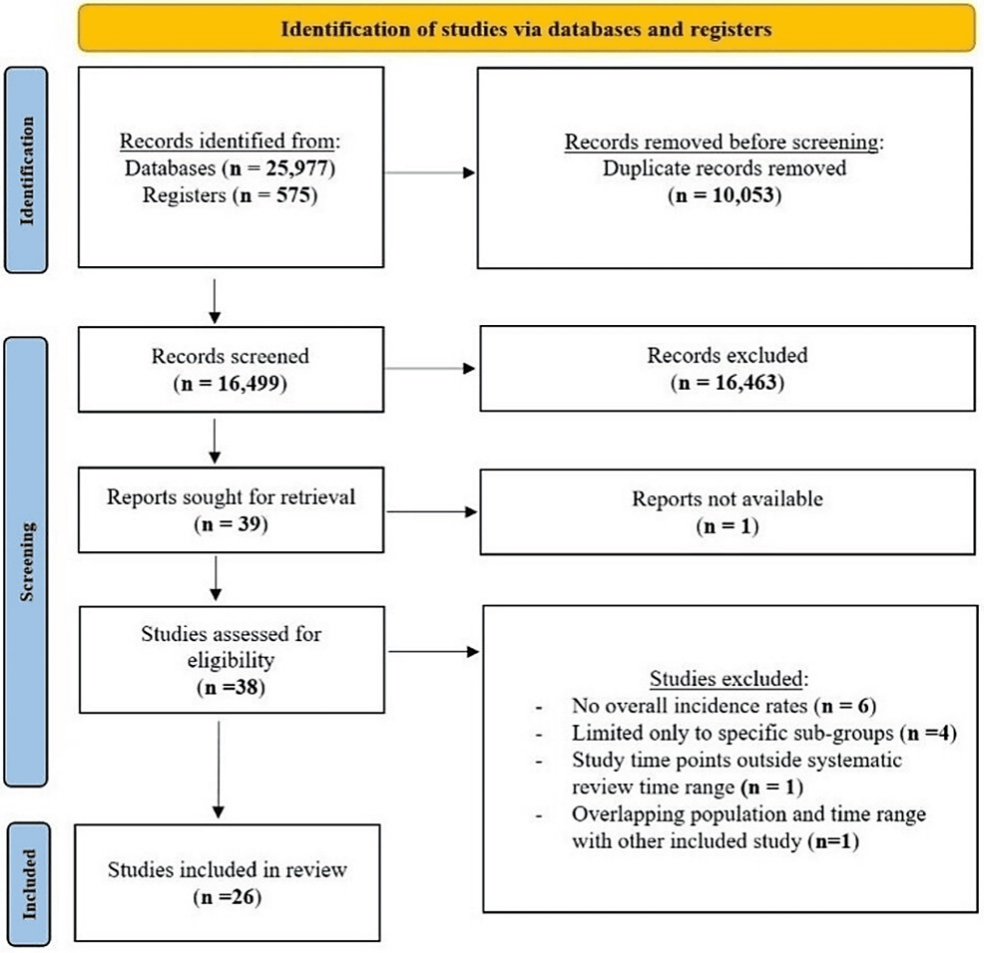
Flow diagram of study selection per 2020 PRISMA guidelines PRISMA: Preferred Reporting Items for Systematic Reviews and Meta-Analyses

Figure 1 is a flow diagram showing the included studies as per the Preferred Reporting Items for Systematic Reviews and Meta-Analyses (PRISMA) 2020 guidelines [34].

**Figure 2:**
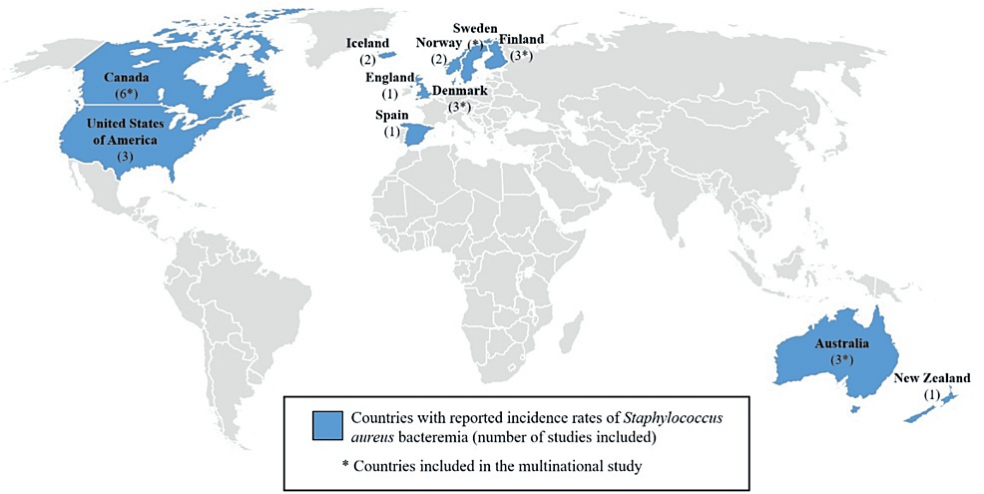
Geographical representation of the 11 countries included in the systematic review

The highest number (n=6) of studies were from Canada; other countries that were included are shown in Figure 2. Of note, 20 (76.9%) of the 26 investigations were regional studies.

Overall Incidence

**Figure 3:**
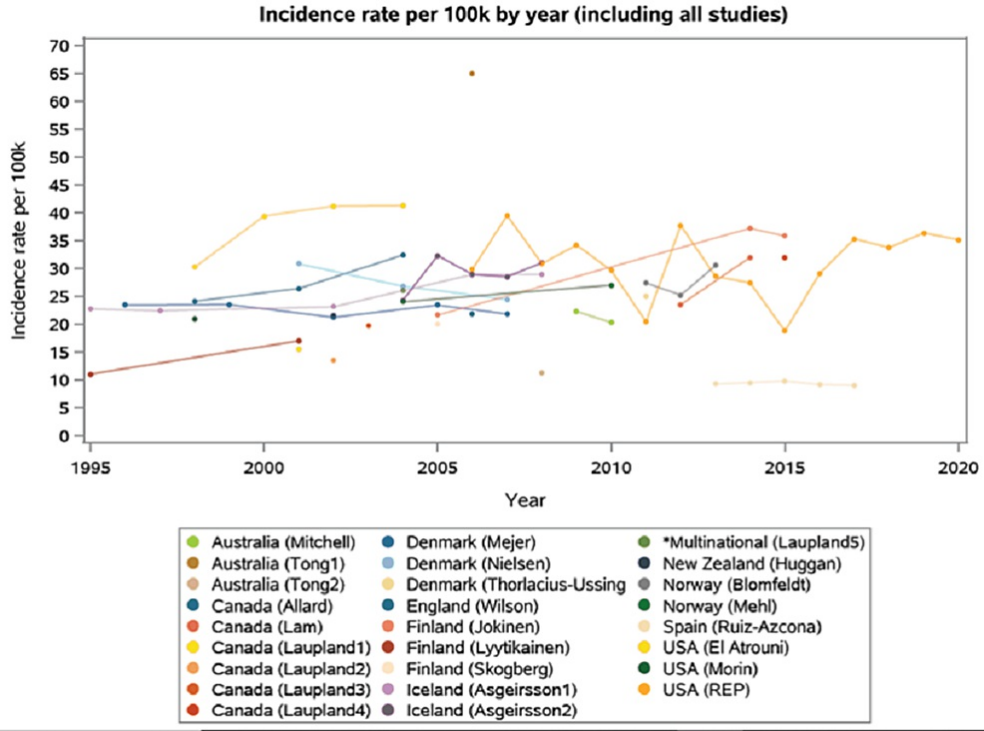
Incidence rate per 100,000/year in included studies *The multinational study included five countries: Australia, Canada, Denmark, Finland, and Sweden

Fourteen studies reported temporal trends in SAB incidences and 12 represented only one time point (Figure 3). 

**Figure 4:**
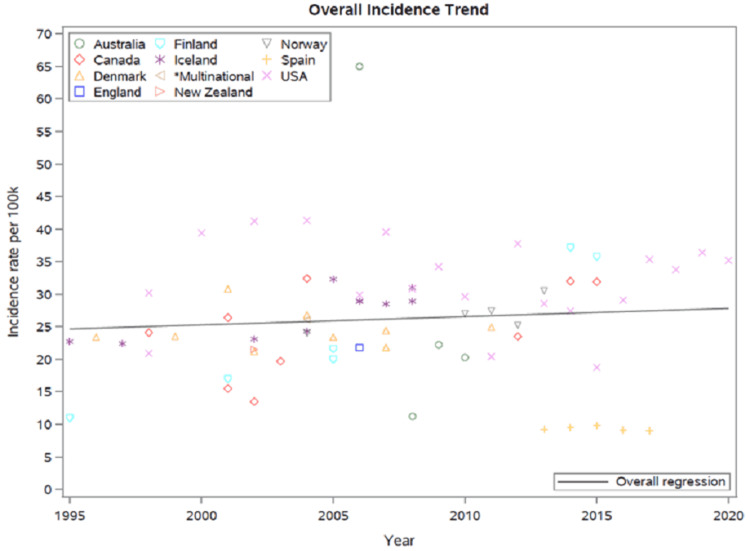
Overall incidence trend of SAB in included studies SAB: *Staphylococcus aureus* bacteremia *The multinational study included five countries: Australia, Canada, Denmark, Finland, and Sweden.

Trends across all included studies demonstrated high variability for geographic regions and calendar years with a SAB incidence ranging from 9.3 to 65 cases/100,000/year. While some showed an increasing incidence of SAB, others revealed decreasing or even unchanging rates over time. Overall, there was no change in the incidence trend across all studies during the past two decades (Figure 4). 

Patient Demographics

The median age of patients with SAB ranged from 62 to 72 years [13,15,20-22,25,27,28,31,32], with the oldest populations from Olmsted County in Minnesota (MN), USA and Oslo, Norway [18,27]. In most of the countries, the rates of SAB were rising with increasing age [8,10,12,14,15,20,26-28,31,32]. However, Ruiz-Azcona et al. reported higher SAB rates in the age range between 45 and 64 years and lower rates in patients aged 65 years and older in Spain [19]. When comparing the two periods of time from 1995 to 1997 and 2004 to 2006, Mejer et al. demonstrated an increase in the proportion of cases in patients older than 75 years and a decrease in the proportion of cases aged one to 55 years [8]. During the past two decades, more than half of SAB cases reported occurred in men [11,13,15,17,18,20-22,25,27,31,32]. Moreover, higher rates of SAB were reported in men than in women in Norway, Denmark, Finland, and Spain [8,10,12,14,19].

In the Danish study by Mejer et al., the proportion of SAB patients with a Charlson comorbidity index (CCI) >0 increased from 1995 to 2008 and the majority of this population had a CCI of 1-2 (39.1%) [8]. In addition, another Danish study restricted to Funen County showed that most of the SAB patients had no comorbidities [9]. However, the majority of the Danish population according to Thorlacius-Ussing et al. had a CCI >3 [10], similar to the SAB populations in Quebec, Canada, and Oslo, Norway [18,20]. Three-fourths of the Canadian population in Calgary from 2000 to 2006 had significant chronic comorbid conditions and/or alcohol use disorder [23]. Compared to SAB patients with no comorbidities, there was increased mortality with increased CCI scores [10].

The prevalence of diabetes mellitus in patients with SAB ranged from 13% to 38% with the highest numbers observed in the USA [16,24,26,27,29]. Moreover, two Canadian studies demonstrated the relative risk of developing SAB in patients with diabetes mellitus was seven to 10.6 [23,24]. The percentage of SAB patients undergoing hemodialysis in the Icelandic and Australian populations ranged between 7.5% and 10.5% [16,30]. However, El Atrouni et al. reported a slightly higher (18.6%) percentage of patients with end-stage renal disease in Olmsted County, MN [27]. A Canadian investigation demonstrated a relative risk of developing SAB in patients undergoing hemodialysis of 360 [23]. According to Tong et al., 33.9% of SAB cases in Australia from 2007 to 2010 were device-related [30]. Ten percent of SAB patients from Calgary, Canada had a prosthetic joint and 5.6% had a permanent pacemaker or implantable cardioverter-defibrillator (ICD) [24].

Only six studies from Australia, Iceland, the USA, and Canada reported percentages of injection drug use (IDU) in their populations [24,26,27,29,30]. The highest number (28%) was observed by Morin et al. in Connecticut, USA in 1998 [26]. However, El Atrouni et al. described a lower percentage (1.6%) in Olmsted County, MN from 1998-to 2005 [27], which was comparable to that reported in Australian and Icelandic studies (0.74% to 5.7%) [16,29,30]. In addition, Lam et al. noted that 18.7% of the population was abusing substances and was not limited to IDU [24].

Based on the New Zealand deprivation index, the least deprived patients had a significantly lower incidence rate of SAB compared to the most deprived [32].

Staphylococcus aureus Bacteremia Characteristics

The highest percentages of methicillin-resistant *Staphylococcus aureus* (MRSA) causing SAB were reported in Canada and the USA (9% to 32%) [20,24-27]; whereas the lowest percentages were described in Norway, Iceland, Denmark, and New Zealand (0.4% to 1.7%) [10,15,18,32]. Two studies from Australia reported different percentages of MRSA in SAB depending on the region (24% in the Northern Territory and 10% in Tasmania) [29,31]. Jokinen et al. reported decreased incidence rates of SAB caused by MRSA in Pirkanmaa County, Finland during the period 2005 to 2015 [14].

The majority of SAB cases from Iceland and Canada were nosocomial (39% to 46%) [15,23], from Norway, USA, and Denmark healthcare-associated (42% to 59%) [8,18,27], and from New Zealand and Australia community-acquired (58% to 64%) [31,32]. In Pirkanmaa County, Finland, the number of healthcare-acquired and community-acquired SAB increased from 2005 to 2015 [14]. Ruiz-Azcona et al. demonstrated an incidence rate of 18.87 per 100,000 inhabitants/year for nosocomial SAB [19].

The most common infection sources of SAB were intravascular catheters and skin and soft tissue infections [20,23,29,30], although four studies from Norway, Iceland, USA, and Canada reported that the majority of their observed SAB did not have an identified source of infection [16,18,24,27]. Other sources were also identified, including infective endocarditis, bone or joint, and respiratory tract infections [16,20,23,24,27,30].

Mortality

The majority of included studies reporting annual mortality rates of SAB in their populations during the past two decades were from Europe [10,12,14-18], with numbers ranging from two to 7.6 per 100,000 inhabitants per year [10,12,14-18,23,32]. The two studies from Iceland demonstrated a gradual decrease in their annual SAB death rates during the periods of 1995 to 2008 and 2003 to 2008 [15,16].

The 30-day mortality rates of SAB ranged from 18% to 29% [10,18,20,25], with the highest number observed in Quebec, Canada from 1997 to 1999 [20]. There was rising 30-day mortality with age [18,25,32], with increasing underlying comorbidities including immunosuppression [18,20,25], MRSA compared to methicillin-susceptible *Staphylococcus aureus* (MSSA) [24], and with healthcare-associated (HCA)-SAB compared to care-associated (CA-SAB) [18,25]. In two studies, women with SAB had higher 30-day mortality compared to men [18,20], whereas one study reported higher mortality rates in men [17]. Also, when grouped by potential sources of SAB, mortality was the highest in patients with pneumonia or infective endocarditis followed by an unknown source of infection [18,20,25]. Interestingly, there was a lower 30-day fatality rate when infectious diseases specialists were consulted [25].

Discussion

The current systematic review represents a contemporary review of SAB incidence in 26 geographical population-based investigations over two decades and demonstrates the striking burden of disease due to SAB. The incidence of SAB ranged from 9.3 to 65 cases/100,000/year. The highest SAB incidence appeared to be an outlier; it was seen in an aboriginal population of the Northern Territory of Australia and was likely due to unique risk factors associated with SAB [29]. This considerable variability in SAB incidence rates between geographic regions and calendar year is likely due to changes in population demographics, socioeconomic status, cultural influences, clinical practices, including those related to infection prevention and control, and surveillance processes. While some studies showed an increased incidence of SAB, others revealed decreasing rates over time. Overall, SAB incidence trends observed during the study period were stable.

The most recent systematic review of population-based studies was conducted in 2013 and assessed both overall bacteremia incidences and SAB incidence more specifically [6]. It showed high variability in rates according to the geographic location with an overall SAB incidence rate of 25 per 100,000 annually. Moreover, this review did not provide overall incidence trends of SAB or demographic data regarding the SAB population.

North America had higher percentages of MRSA causing SAB [20,24,26,27] as compared to Iceland and Denmark [10,15,32]. In the early 21st century, patients in Northern European countries were undergoing aggressive screening for MRSA with eradication in colonized patients [35]. The implementation of this new policy to search and destroy MRSA could account for the low percentages of MRSA in the region [10,15].

During the past two decades, the highest rates of SAB were observed in the Northern Territory of Australia’s population [29]. When stratified by ethnicity, Aboriginal patients had a six-times higher rate of SAB than non-Aboriginal patients. This significantly correlates with socioeconomic disadvantages related to access to healthcare and education, as well as housing and employment in this population as decreasing socioeconomic status was linked to an increased risk of SAB [32,36].

A Danish study reported high incidence rates of SAB in an HIV-infected population that had IDU from 1995 to 2007 [37]. The percentage of IDU in a SAB population was the highest (28%) in Connecticut, USA in 1998 [26]. In contrast, a much lower (1.6%) percentage was reported more recently in MN, USA [27]; of course, this could be an underestimation since IDU can be underreported, or related to geographic heterogeneity related to opioid use in more rural areas of the USA [38]. The highest percentages (9% to 32%) of MRSA causing SAB were also observed in the USA [26,27], which could be explained by the rising IDU prevalence since one-fourth of SAB caused by MRSA were IDU-related in the USA [39].

One-third of SAB cases were device-related [30]; the increasing utilization of indwelling foreign devices including vascular catheters, and orthopedic prostheses in clinical practice is a well-established risk factor associated with SAB [40]. Also, patients undergoing hemodialysis have an increased SAB incidence rate, particularly when chronic indwelling vascular catheters are used for hemodialysis access [23,41]. Of note, patients are most at risk during the first three months of hemodialysis [41].

There were several limitations to this systematic review despite its rigorousness. First, while multinational, studies included in our review do not represent a global aspect of SAB incidence since it does not include coverage of many geographic regions, namely Africa, Asia, and South and Central America, highlighting the need for population-based investigations from regions worldwide. Second, while our systematic review included all studies, regardless of geographic location, the inclusion of non-English language articles could have enhanced the likelihood of finding data from other countries, particularly developing countries. Third, not all included studies provided consistent data regarding their population demographics, comorbidities, and mortality rates. Fourth, some studies reported incidence trends of SAB, whereas others reported only one time point; this may have contributed to the absence of clear change in SAB incidence trends overall.

## Conclusions

The current systematic review represents the most contemporary review of SAB incidence in 26 geographical population-based investigations during the past two decades. It shows an overall stable SAB incidence in the 11 countries included ranging from 9.3 to 65 cases/100,000/year. Multiple factors, both pros, and cons are likely responsible for the overall stable SAB incidence. Some of these factors vary in geographic location and prompt additional investigations from countries not included in the current review are required so that a more global characterization of SAB is defined.

## Figures and Tables

**Figure 5:**
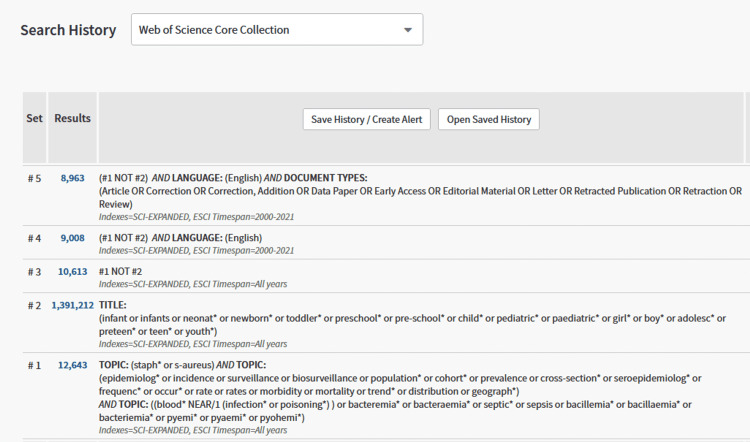
Web of Science core collection via Clarivate Analytics (Science Citation Index Expanded 1975+ and Emerging Sources Citation Index 2015+)

## Appendices

**Table 2:** The number of citations per database and registers MEDLINE: Medical Literature Analysis and Retrieval System Online

Databases & Registers	# of initial hits
Central (clinical trial register)	575
Embase	10,880
MEDLINE	6,134
Web of Science	8,963
Duplicates	10,053
Totals	16,499

**Table 3:** Cochrane Central Register of Controlled Trials (CCTR) via Ovid (1991+)).

#	Query	Results from 2 Sep 2021
1	(staph* or s-aureus).ab,hw,ti.	6,268
2	(epidemiology* or incidence or surveillance or biosurveillance or population* or cohort* or prevalence or cross-section* or seroepidemiology* or frequency* or occur* or rate or rates or morbidity or mortality or trend* or distribution or geography*).ab,hw,ti.	830,218
3	((blood* adj1 (infection* or poisoning*)) or bacteremia* or bacteraemia* or septic* or sepsis or bacillemia* or bacillaemia* or bacteriemia* or pyemi* or pyaemi* or pyohemi*).ab,hw,ti.	19,172
4	1 and 2 and 3	912
5	(infant or infants or neonate* or newborn* or toddler* or preschool* or pre-school* or child* or pediatric* or paediatric* or girl* or boy* or adolesc* or preteen* or teen* or youth*).m_titl.	143,148
6	4 not 5	768
7	limit 6 to yr="2000 -Current"	575

**Table 4:** Embase via Ovid (1974+).

#	Query	Results from 2 Sep 2021
1	exp Staphylococcus aureus/ or exp Staphylococcus infection/	207,435
2	(staph* or s-aureus).ab,dj,kw,ti.	203,208
3	1 or 2	273,335
4	exp epidemiological data/ or exp epidemiology/	4,555,005
5	exp trend study/	39,019
6	(epidemiolog* or incidence or surveillance or biosurveillance or population* or cohort* or prevalence or cross-section* or seroepidemiolog* or frequenc* or occur* or rate or rates or morbidity or mortality or trend* or distribution or geograph*).ab,dj,kw,ti,fx,sh.	12,961,416
7	4 or 5 or 6	13,631,494
8	3 and 7	111,463
9	bacteremia/ or staphylococcal bacteremia/ or exp bloodstream infection/ or sepsis/ or ((blood* adj1 (infection* or poisoning*)) or bacteremia* or bacteraemia* or septic* or sepsis or bacillemia* or bacillaemia* or bacteriemia* or pyemi* or pyaemi* or pyohemi*).ab,kw,ti.	353,305
10	8 and 9	23,047
11	limit 10 to English language	21,167
12	limit 11 to yr="2000 -Current"	18,670
13	exp juvenile/ not adult/	2,363,604
14	12 not 13	15,696
15	limit 14 to conference abstract	4,299
16	14 not 15	11,397
17	exp animal/ not exp human/	4,829,043
18	16 not 17	10,880

**Table 5:** MEDLINE via Ovid (1946+ and Epub ahead of print, in-process and other non-indexed citations and Ovid MEDLINE(R) Daily) MEDLINE: Medical Literature Analysis and Retrieval System Online

#	Query	Results from 2 Sep 2021
1	exp Staphylococcus aureus/ or exp Staphylococcal Infections/	115,177
2	(staph* or s-aureus).ab,kw,ti.	151,134
3	1 or 2	179,717
4	exp Morbidity/ or exp Mortality/	962,601
5	(epidemiolog* or incidence or surveillance or biosurveillance or population* or cohort* or prevalence or cross-section* or seroepidemiolog* or frequenc* or occur* or rate or rates or morbidity or mortality or trend* or distribution or geograph*).ab,hw,kw,ti,fx.	9,233,452
6	4 or 5	9,287,700
7	3 and 6	65,570
8	exp Bacteremia/ or Sepsis/ or ((blood* adj1 (infection* or poisoning*)) or bacteremia* or bacteraemia* or septic* or sepsis or bacillemia* or bacillaemia* or bacteriemia* or pyemi* or pyaemi* or pyohemi*).ab,kf,ti.	196,177
9	7 and 8	11,385
10	limit 9 to English language	9,883
11	limit 10 to yr="2000 -Current"	7,202
12	(exp CHILD/ or Adolescent/) not exp ADULT/	1,500,200
13	11 not 12	6,528
14	exp Animals/ not Humans/	4,879,221
15	13 not 14	6,134
